# Direct Band Gap Gallium Antimony Phosphide (GaSb_x_P_1−x_) Alloys

**DOI:** 10.1038/srep20822

**Published:** 2016-02-10

**Authors:** H. B. Russell, A. N. Andriotis, M. Menon, J. B. Jasinski, A. Martinez-Garcia, M. K. Sunkara

**Affiliations:** 1 Department of Chemical Engineering and Conn Center for Renewable Energy Research University of Louisville, Louisville, KY, USA; 2 Center for Computational Sciences, University of Kentucky, Lexington, KY, USA; 3 Department of Physics and Astronomy, University of Kentucky, Lexington, KY, USA; 4 Institute of Electronic Structure and Laser (IESL), Foundation of Research and Technology-Hellas (FORTH), Heraklion, Crete, Greece

## Abstract

Here, we report direct band gap transition for Gallium Phosphide (GaP) when alloyed with just 1–2 at% antimony (Sb) utilizing both density functional theory based computations and experiments. First principles density functional theory calculations of GaSb_x_P_1−x_ alloys in a 216 atom supercell configuration indicate that an indirect to direct band gap transition occurs at x = 0.0092 or higher Sb incorporation into GaSb_x_P_1−x_. Furthermore, these calculations indicate band edge straddling of the hydrogen evolution and oxygen evolution reactions for compositions ranging from x = 0.0092 Sb up to at least x = 0.065 Sb making it a candidate for use in a Schottky type photoelectrochemical water splitting device. GaSb_x_P_1−x_ nanowires were synthesized by reactive transport utilizing a microwave plasma discharge with average compositions ranging from x = 0.06 to x = 0.12 Sb and direct band gaps between 2.21 eV and 1.33 eV. Photoelectrochemical experiments show that the material is photoactive with p-type conductivity. This study brings attention to a relatively uninvestigated, tunable band gap semiconductor system with tremendous potential in many fields.

The production of clean fuels by utilizing solar energy through photoelectrochemical water splitting is one of the most important milestones on the road to a sustainable energy future^1^,^2^. Toward this objective, new semiconductor materials with appropriate band gap, band edge placement, electron mobility and chemical stability are still greatly needed to enable efficient solar hydrogen production^3^. Almost all singular and binary semiconductor materials have been explored without a clear contender for solar hydrogen generation, yet most ternary and quaternary materials remain relatively uninvestigated. III-V semiconductors are to date the best photoabsorber materials known. Devices containing III-V photoabsorbers currently hold the records in both photovoltaic cells (GaInP/GaAs, GaInAsP/GaIn multijunction cell ~ 46% efficiency)^4^ and photoelectrochemical water splitting (GaInP/GaAs monolithic cell ~12.4% efficiency)^5^. With the exception of cuprous oxide^6^, III-V photoabsorbers have attained higher photocurrent densities under unbiased condition than any other class of photoabsorber materials^7^,^8^,^9^.

Of particular interest is GaP, a well-known semiconductor with an indirect band gap of 2.3 eV, band edge alignment desirable both for photoelectrochemical water splitting as well as CO_2_ reduction^10^,^11^,^12^, and is stable as a photocathode for photoelectrochemical water splitting under reducing conditions^13^,^14^. GaP is also desirable due to its low lattice mismatch with silicon of 0.37%, which could allow for economical growth of GaP epi-layers using silicon substrates^15^,^16^,^17^. However, GaP has an indirect band gap which requires a relatively large absorption depth in comparison to the relatively low minority carrier diffusion length^18^ and poor surface kinetics^13^.

Ternary III-V alloys have received a lot of attention due to their ability to exhibit tunable band gaps by changing compositions. In fact, the current state of the art water splitting device is GaInP_2_, a III-V ternary alloy that combines an indirect band gap GaP with direct band gap InP to create a 1.83 eV direct band gap semiconductor^5^. Indirect to direct transitions are seen in several other GaP ternary alloys including alloys containing Nitrogen, Arsenic and Indium. GaPN has been shown to exhibit an indirect to direct transition with compositions as low as GaP_0.9994_N_0.0006_^8^,^19^. GaPAs undergoes a direct transition at a composition of GaP_0.44_As_0.56_^20^. InGaN, GaSbN, GaAsPN and GaPN have each been researched for water splitting but each have their setbacks^8^,^9^,^21^,^22^.

Also of interest is Gallium Antimonide (GaSb), another III-V semiconductor with a direct band gap of 0.725 eV^23^. The alloying of GaSb and GaP systems could allow for band gap energy tuning, indirect to direct transition and band edge straddling for both hydrogen and oxygen evolution reactions. As evident from Fig. 1, the band edge positions for GaP straddle both the hydrogen evolution reaction (HER) and oxygen evolution reaction (OER) while GaSb only straddles the HER at pH = 7. Thus, under the low Sb composition regime of GaSb_x_P_1−x_, there are ternary alloy compositions that should straddle both HER and OER. Furthermore, if the direct to indirect band gap transition occurs in this low Sb incorporation regime similarly to GaPN, then an alloy composition of GaSb_x_P_1−x_ with a direct band gap capable of driving the water splitting reaction unbiased could be achieved.

Few studies exist for ternary alloys involving GaSb_x_P_1−x_. Initially, it was thought impossible to grow epitaxial layers of ternary alloys due to the miscibility gap between GaP and GaSb across the entire range of compositions at typical growth temperatures of 530C^24^. GaSb_x_P_(1−x)_ epilayers were still eventually synthesized by Stringfellow *et al.* using organometalic vapor phase epitaxy^25^. The layers were grown in several compositions ranging from x = 0.14 to x = 0.93 that exhibited cold photoluminescence responses at approximately 0.8 eV, 1.3 eV, 1.4 eV and 1.6 eV, but it was not clear whether any of the above compositions allowed for direct band gap transitions. It has been further researched for its ability to form a Schottky diode with InP^26^, as a Vertical Cavity Emitting Surface Laser^27^, using the Stranski Krastananov mode growth on GaP^28^. Electron transport in GaPSb films has also been investigated^29^.

Prior studies have not clearly indicated whether there was any sort of direct band gap transition for GaSbP alloys. In addition, there are no studies reported about the use of GaSb_x_P_(1−x)_ alloys toward solar fuels research and the band edge alignment with respect to hydrogen and oxygen evolution reactions. Here, in this study, both the theoretical computations using density functional theory with the Hubbard correction parameter (DFT + U) (see section Method: Theoretical) for GaP_(1−x)_Sb_x_ in the low Sb regime in the bulk form and the experimental synthesis of GaP_(1−x)_Sb_x_ alloys as nanowires with average Sb incorporation from x = 0.06 to x = 0.12 are investigated. The GaP_(1−x)_Sb_x_ nanowires are synthesized using a reactive vapor transport technique involving solid precursors (Ga & Sb metals) and a gas phase precursor for phosphorous in a microwave plasma reactor. The GaSb_x_P_1−x_ NWs were characterized using transmission electron microscopy (TEM), X-ray Diffraction (XRD), UV-Vis diffuse reflectance spectroscopy, photoluminescence, Raman spectroscopy and photoelectrochemical water splitting activity testing. Note, the composition parameter, x, is referring to the atomic concentration of Sb with respect to phosphorus in GaSb_x_P_(1−x)._

## Results and discussion

**Figure 2:**
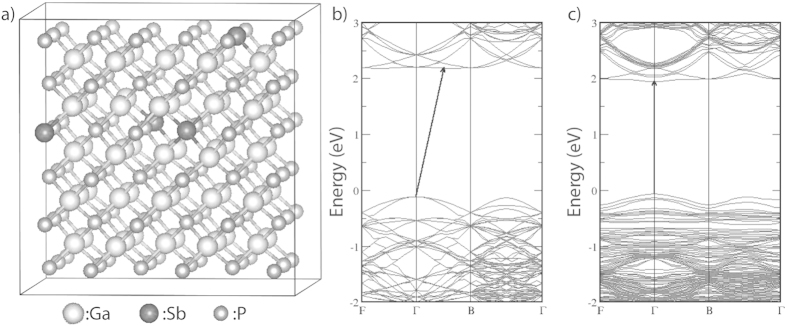
(**a**) A 216 atom Supercell of GaP_0.963_Sb_0.037_ optimized using DFT calculations and a comparison of density of states diagrams for (**b**) GaP and (**c**) GaP_0.963_Sb_0.037_ showing the direct optical band gap transition with 1.8% Antimony incorporation.

The theoretical work modeled bulk GaP with zinc blende structure using a relatively large, 216 atom (2 × 2 × 1 unit cell) supercell with periodic boundary conditions. The size of the supercell is large enough to allow for random distribution of Sb atoms. GaSb_x_P_(1−x)_ structures are obtained by substituting P atoms with Sb (isovalent substitution). In each case considered, both the cell volume and the individual atomic positions have been fully optimized without any symmetry constraints. The optimized structure of the alloy with composition for 4 incorporations of Sb (x = 0.037) is shown in Fig. 2a.

**Figure 3:**
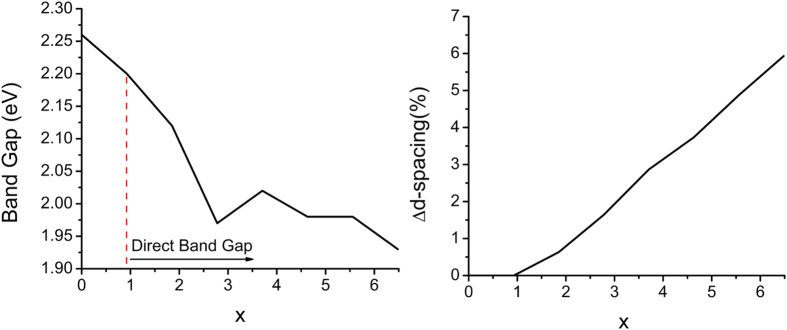
(**a**) Change in band gap for antimony incorporation up to x = 0.065 Sb and (**b**) percent change in d-spacing for up to 3.2% antimony incorporation. Both plots were obtained from DFT calculations.

The alloying of GaP by Sb introduces impurity (*spd*-hybridized Sb-related) states at the gap edges. Those at the valence band maximum (VBM) are more pronounced (not shown). The Sb-related states lead to significant modifications in the energy bands. As evident from Fig. 2b,c, the Sb-states induce a strong band repulsion at the conduction band minimum (CBM) of the GaP bands that lead to the lowering of the bands at the Γ-point which results in turning the indirect gap into a direct one. On the other hand, new Sb bands are introduced at the VBM. The new bands lead to a band repulsion that lifts the degeneracy of the GaP-bands at the Γ-point, which in turn, leads to a small reduction in the energy gap. This trend in band gap reduction is seen for increasing Sb incorporation alongside a corresponding increase in d-spacing (Fig. 3).

The estimated band gap values can depend on the configuration of the distributed Sb impurities. So there will be an error bar associated with each calculated point of the data. Unfortunately the estimation of the error bars is computationally prohibitive. For this reason, the plot of band gap as a function of x is best represented using a least square polynomial function instead of simply connecting data points. As long as no Sb-Sb interactions are present (i.e. the Sb atoms are far from each other) a linear variation of band gap vs. x could be expected as this results from the appearance of Sb states in the band edges and band repulsion effects. Both of these effects however, may not be simply proportional to x for large enough x. Nevertheless, in the range of the studied Sb concentrations, (maximum 3–4 Sb atoms in 108 set of P-atoms) no Sb-Sb interactions should be expected and therefore the effect from each Sb atom to be additive.

**Figure 4:**
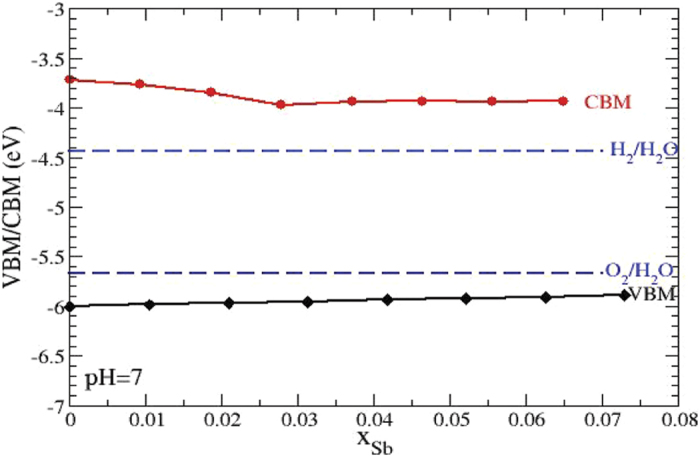
Valence band maximum and conduction band minimum positions calculated by the generalized Harrison’s approach as a function of Sb concentration in Ga(Sb)P at pH = 7. Also included are the redox potentials of H_2_O.

Another important study is to estimate the band edge location with respect to vacuum. Recall that the ionization potential (IP) of a (non-polar) semiconductor specifies the energy of the VBM with respect to vacuum^30^. Very recently, the same group reported accurate IP estimation for a set of non-polar semiconductors in good agreement with experiment^31^. For GaP and GaSb, the corresponding Ionization Potential values are in the ranges [5.95–6.01] eV and [4.70–4.94] eV. In view of these, the VBM of GaP is at approximately 6.0 eV below the vacuum level while the CBM is approximately at the energy: VBM + E_gap_. In order to find the variation of the VBM of GaP as the Sb concentration, x, is varied, the recently proposed^32^, virtual crystal approximation (VCA) extension of Harrison’s method for evaluating the VBM was used^33^,^34^. The values so obtained are shifted in such a way that the VBM-value for x = 0 (i.e., that for GaP) coincides with values that are negative of the IP of GaP. In order to find the CBM as a function of Sb-concentration, the band gap values for each concentration are added to the corresponding VBM at the same concentration. A plot of VBM and CBM with respect to vacuum in terms of Sb-concentration is presented in Fig. 4 in comparison to the hydrogen evolution reaction and oxygen evolution potentials. As can be seen from the position of these bands, theoretical results predict the straddling of the HER and OER for up to x = 0.074 incorporation of Sb.

GaSb_x_P_(1−x)_ NW samples were grown using reactive vapor transport in a microwave plasma reactor. Ga and Sb metals were placed on opposite sides of a graphite susceptor with clean substrates placed in between. The reactor was pressurized with H_2_ to a chamber pressure of 100 torr and microwave plasma was held over the susceptor at powers between 800 W and 1000 W. Upon stabilizing the plasma at the target operating power, ditertbutyl-phosphine (DTBP) was fed into the reactor. The reaction was allowed to take place for 2 hours.

**Figure 5:**
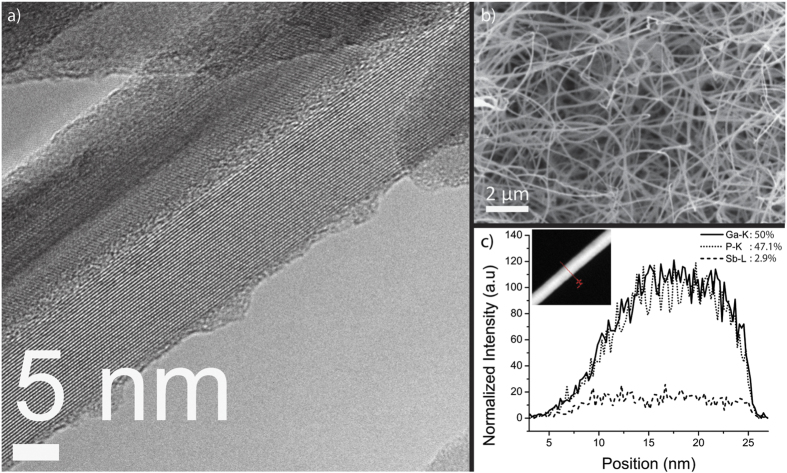
(**a**) A high resolution transmission electron micrograph of a GaSb_x_P_(1−x)_ nanowire; (**b**) A scanning electron micrograph of GaSb_x_P_(1−x)_ Nanowires; and (**c**) An EDS line scan for composition using transmission electron microscopy showing Ga, Sb and P concentration across a radial cross section of the GaSb_x_P_(1−x)_ nanowire from part a.

The resulting GaSb_x_P_(1−x)_ samples exhibited nanowire morphologies from 20 nm up to 1 μm in diameter and several microns in length as shown with transmission electron microscopy and scanning electron microscopy (SEM) images in Fig. 5a. The mechanism for growth of the resulting nanowires is similar to that of self-catalyzed growth of nanowires with Ga droplets leading the growth. A cross section of the GaSb_x_P_(1−x)_ sample was exposed by cleaving the sample and investigated using Energy Dispersive X-Ray Spectroscopy (EDS). See Fig. 1 in supplemental information. At the interface between the silicon and film growth, only Ga and Sb are present at a 1:1 atomic ratio as a thin film. The formation of GaSb at the growth interface is expected due to the operation of the plasma with only Ga and Sb present before the addition of the phosphorous precursor, Di-Tertiary Butyl Phosphene. The majority of the sample above the interface exhibits the presence of phosphorus addition to Ga and Sb.

**Figure 6:**
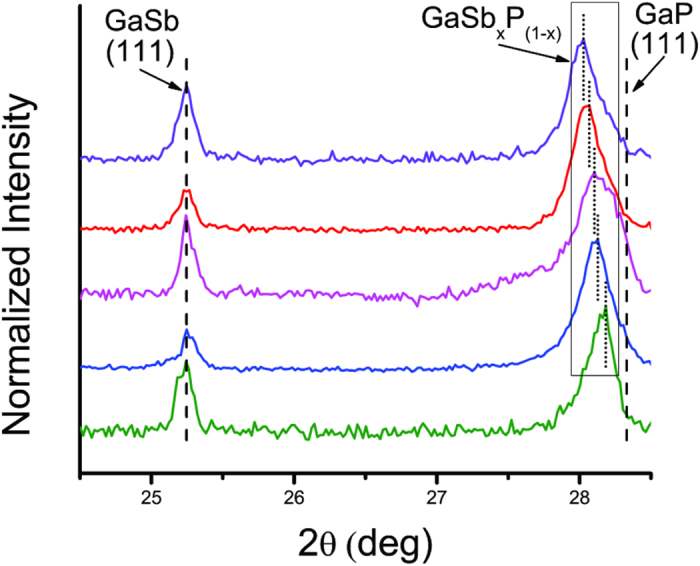
X-Ray Diffraction pattern of Ga_Sb__x_P_(1−x)_ samples indicating alloy compositions. Individual concentrations were determined to be x_Sb_ = 0.121, 119, 106, 100 and 0.059 (from top to bottom).

X-Ray Diffraction (XRD) of five different GaSb_x_P_(1−x)_ samples show major peaks for GaSb as well as a set of peaks similar to GaP, but shifted toward those of GaSb (Fig. 6). The XRD peak at 25.3 degrees indicates the presence of GaSb which is expected to be formed in the initial stages of growth prior to the introduction of phosphorous precursor. For the (111) reflection, the observed Bragg angle shifts toward lower angles from 28.3 degrees expected for pure GaP (111); this peak shift can be used for estimating the Sb composition in the GaSb_x_P_(1−x)_ alloy using Vegard’s law. The Sb compositions based on XRD peak shift for various samples were determined to be between x = 0.06 and x = 0.12.

**Figure 7:**
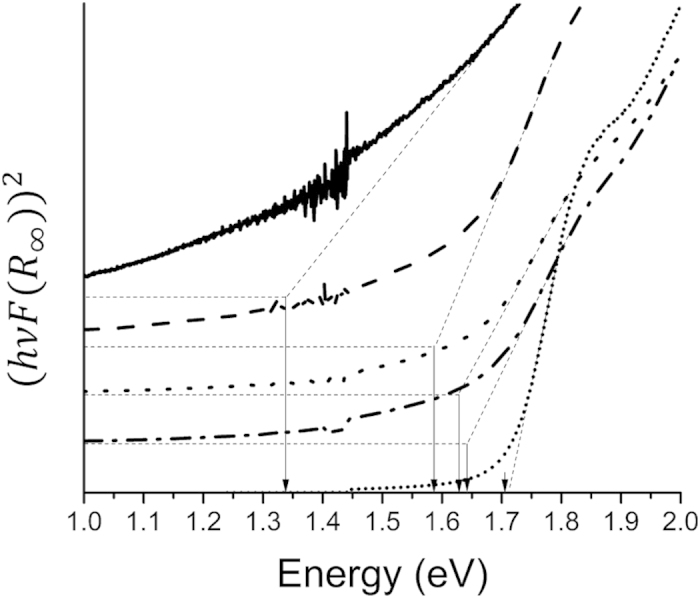
Tauc plot analysis for the direct allowed transition from UV-Vis diffuse reflectance for the GaSb_x_P_(1−x)_ samples indicating direct band gap transitions from 1.33 eV up to 1.72 eV.

Diffuse reflectance ultraviolet-visible spectroscopy (UV-Vis) measurements for the above five GaSb_x_P_(1−x)_ samples were performed. Tauc plots for the direct allowed transition indicating direct band gaps between 1.33 and 1.72 eV are shown in Fig. 7. The measurement utilizes absorption over an area exceeding 1 cm^2^ such that the estimated optical band gap is an average value. Most importantly, the UV-Vis data and its corresponding analysis using Tauc plots did not show the presence of binary III-V phases (GaSb or GaP) thus confirming the presence of primarily ternary III-V alloys.

In all of the samples, there is a spatial variation for compositions of ternary alloys. In some samples, the variation for Sb composition can be anywhere from x = 0.02 to x = 0.24 over a two-inch area. However, there is little or no (less than x = 0.01) variation in Sb composition along the length of each nanowire. See Fig. 2 in supplemental information. High resolution TEM images in Fig. 5b reveal high single crystallinity for GaSb_x_P_(1−x)_ nanowires with no defects and no amorphous sheath. Increased amount of stacking faults were observed in nanowires with high amounts of Sb composition. TEM EDS line-scan profiles shown in Fig. 5c radially across the nanowire diameter show similar profile curves for Ga, P, and Sb indicating true alloy formation with no segregation. Furthermore, no binary (GaSb or GaP) nanowires were observed.

**Figure 8:**
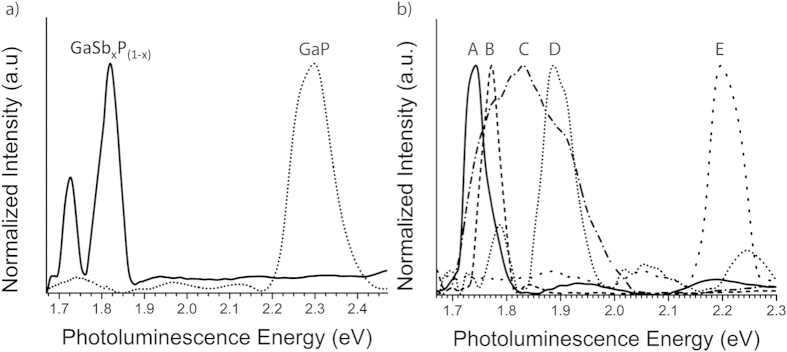
(**a**) Visible photoluminescence spectra at liquid nitrogen temperature (77 K) for GaSb_x_P_(1−x)_ alloy containing peaks at 1.73 and 1.84 eV and a peak at 2.3 eV for GaP control sample; (**b**) Visible photoluminescence from various regions (**A**–**E**) of GaSb_x_P_(1−x)_ nanowire film sample at room temperature that exhibit peaks at 1.74 eV (**A**), 1.77 eV (**B**), 1.84 eV (**C**), 1.89 eV (**D**), and 2.21 eV (**E**).

Micro-photoluminescence (PL) measurements were performed using a spot size on the order of few microns on the 1.72 ev band gap GaSbP sample at liquid nitrogen (77 K) and room temperature (300 K). At 77 K, a GaSb_x_P_(1−x)_ nanowire film sample showed responses at 1.73 and 1.82 eV. A GaP control sample at this temperature showed a response at 2.3 eV as expected and no response at 300 K due to the indirect nature of band gap. From the cold PL measurements at 77 K shown in Fig. 8a, it can be inferred that there is no pure GaP phase present in the measured GaSb_x_P_(1−x)_ nanowire film region. At liquid nitrogen temperatures, non-radiative recombination is minimized and electrons are trapped in the GaP conduction band edge only allowing them to emit PL from that band. Thus, if there was binary GaP present in the measurement area at 77 K, an emission at ~2.3 eV would be expected. PL measurements at 300 k have yielded varying PL peak positions at various locations indicating different direct band gap values with different compositions for nanowires. The observed PL peak positions ranged from 1.73 eV to 2.21 eV across the same sample as shown in Fig. 8b. The range of band gaps estimated using PL are consistent with optical band gap measurements for this material obtained by UV-Vis spectroscopy. UV-Vis and XRD techniques use averaged information over 1 cm^2^ area while PL spectroscopy’s information is from about few micron square area and TEM data corresponds to individual nanowire.

The room temperature PL peaks could also be due to the presence of impurity states within the band gap of the GaP semiconductor. In order to be sure that these PL responses are not from other impurity donor and acceptor levels, the levels for possible donors and acceptors are reviewed. Silicon acts as a shallow donor (E_c_ − 0.085) when found in a Ga vacancy and a shallow acceptor (E_v_ + 0.21) when found in a Phosphorus vacancy, if emission occurred from this donor to acceptor level, a PL emission of approximately 2 eV would be expected. Oxygen can only be found in Phosphorus vacancies and acts purely as a deep level donor (E_c_ – 0.89) so an approximate emission from this deep state would be expected at 1.41^35^. Three levels of Ga vacancies can exist, all of which act as acceptors, and based upon MS XCK cluster model calculations by Fazzio, Brescansin and Leite have values as follows: V^0^_Ga_ ~ E_V_ + 0.38, V^−1^_Ga_ ~ E_V_ + 0.8, and V^−2^_Ga_ ~ E_V_ + 0.9^36^. If these vacancies were present expected emissions would occur at 1.92 eV, 1.5 eV and 1.4 eV. So, the observed room temperature and cold PL peaks correspond to band edge to band edge transitions and thus represent band gaps for the synthesized GaSb_x_P_1−x_ alloy nanowires.

**Figure 9:**
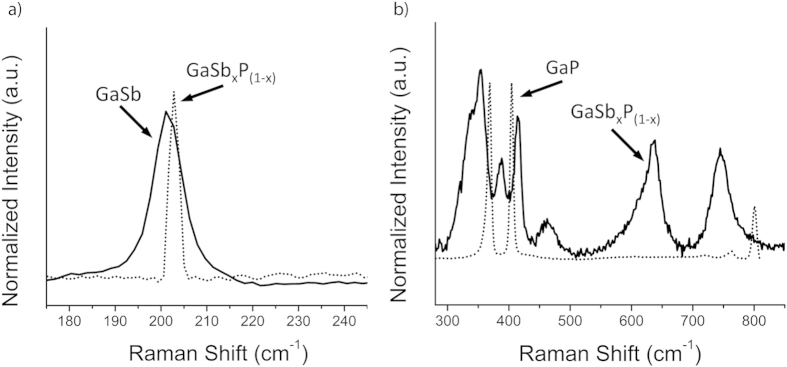
Raman of GaSb_x_P_(1−x)_ against (**a**) GaSb and (**b**) GaP control samples. Major peaks for GaSb_x_P_(1−x)_ are observed at 201 cm^−1^, 356 cm^−1^, 390 cm^−1^, 417 cm^−1^, 464 cm^−1^, 637 cm^−1^ and 750 cm^−1^.

As can be seen in Fig. 9a, the Raman peak observed at 201 cm^−1^ is shifted from that expected for pure GaSb phase. This slight mismatch can be attributed to both the nanocrystalline and ternary nature of the GaSb_x_P_(1−x)_ alloy. Peak shifts could arise from temperature rise within nanowire samples due to laser irradiation^37^. In Fig. 9b, Raman spectra for a pure GaP control sample is presented along with that obtained for a GaSb_x_P_(1−x)_ sample. The data shows a significant peak shift for the two major peaks expected for GaP phase and also an additional peak in that region. Additional peaks at 464, 637 and 750 cm^−1^ are also observed. No previous Raman data exists for the GaSb_x_P_(1−x)_ system. Similarly, additional peaks were observed in dilute ternary GaSb_x_N_1−x_ alloys in addition to those expected for binary phase, GaN^22^. The origin of such peaks needs further investigation.

Preliminary Open Circuit Potential (OCP) measurements show conclusive evidence of p-type conductivity under illumination making it a promising candidate for use as the photocathode in a water splitting cell (Fig. 3a in supplemental information). Three electrode linear sweep voltammetry measurements under chopped illumination show a photocurrent density of approximately 40 μA/cm^2^, however the large cathodic current observed without illumination would suggest that, simultaneously, a corrosion reaction may be happening (Fig. 3b in supplemental information). Unbiased 2 electrode IV measurements were performed on the 1.6 eV band gap GaSbP sample grown on quartz substrate. A photocurrent of approximately 20 μA/cm^2^ was observed (Fig. 3c in supplemental information). Chronoamperometry essays show that the photoactivity remains constant over time indicating it is a result of true water-splitting by GaSb_x_P_(1−x)_.

## Conclusions

Theoretical computations using DFT + U type methodology were used to investigate GaSb_x_P_(1−x)_ alloy system with x ranging from 0 to 0.065. The computations predicted direct optical band gap transition for alloys containing x = 0.0092 or more. Theoretical results also showed that for up to x = 0.065, the band edges straddle HER and OER reactions. GaSb_x_P_(1−x)_ nanowires were obtained using a reactive transport method without the use of any additional catalyst. Electron microscopy studies confirmed single crystallinity for the resulting GaSb_x_P_(1−x)_ alloy NWs with uniform compositions of Ga, Sb and P throughout their lengths. Sb concentrations in these NWs ranged from x = 0.02 to x = 0.24. XRD confirmed the presence of ternary alloys GaSb_x_P_(1−x)_, without the presence of pure GaP phase. UV-Vis diffuse reflectance of various samples confirmed direct band gaps between 1.33 eV and 1.72 eV. Room temperature photoluminescence micro-measurements confirmed direct band gaps for GaSb_x_P_1−x_ nanowire samples and cold photoluminescence (77 K) did not show any luminiscence at 2.26 eV indicating no presence of pure GaP in the sample. Photoelectrochemical linear sweep voltammetry measurements indicated that the GaSb_x_P_1−x_ samples are photoactive. OCP measurements indicated the alloy has an extrinsic p-type response and two electrode voltammetry showed a consistent photoresponse at zero bias indicating these materials straddle the water splitting reaction and show that GaSb_x_P_1−x_ is capable of splitting water as a photocathode in a Schottky type water splitting device. Most importantly, the experimental findings corroborate the DFT + U calculations which predicted that indirect to direct band gap transition occurs even with small amounts of Sb incorporation and the band edges for the resulting alloys straddle the water splitting reaction.

## Methods

### Theoretical

*Ab initio* calculations were performed using the density functional theory in the generalized gradient approximation (GGA) at the Perdew-Burke-Ernzerhof (PBE)^38^ level of approximation for exchange and correlations as implemented in the Vienna Ab-initio Simulation Package (VASP)^39^,^40^,^41^. The projected augmented wave (PAW) potential, is used to describe the core electrons^40^,^41^. To circumvent the well-known “gap problem” in the DFT/GGA formalism, the Hubbard correction parameter, U, was incorporated in the formalism (DFT/GGA + U method)^42^ based upon Dudarev’s approach^43^. After testing for convergence, a 2 × 2 × 1 gamma–centered pack was chosen for **k**-vectors sampling. The same set of k-points were used in the calculation for density of states (DOS). A kinetic energy cutoff of 520 eV was found to be sufficient to achieve a total energy convergence of the energies of the systems to within 1 meV. Gaussian smearing of 0.05 eV was chosen to accelerate the electronic convergence. The optimization of atomic positions (including full cell optimization) was allowed to proceed without any symmetry constraints until the force on each atom is less than 5 meV/Å.

The optimized geometric structures in all cases were obtained using the GGA + U method prior to performing calculations for band structure and DOS. The U values used in the GGA + U formalism are: U_d_(Ga) = 6.5 eV, U_p_(P) = –14.5 eV and U_p_(Sb) = –4.2 eV. All other U values such as U_p_(Ga), U_s_(Ga), U_s_(P) and U_s_(Sb) are set to zero. The U-values have been obtained by fitting to experimental and/or theoretical data for the band gap and, if available, the electron DOS. This GGA + U method was used to calculate the band structure and DOS. All self-consistent calculations were carried out using a convergence criterion of 10^−6 ^eV. The geometric structures in all cases were fully optimized without any symmetry constraints using the GGA + U method prior to performing calculations for band structure and density of states (DOS). The efficiency of the GGA + U approach has been demonstrated in a series of recent reports^21^,^22^,^42^. Sometimes, the fitting approach leads to negative U-values as used and justified in recent reports^44^,^45^,^46^,^47^,^48^,^49^.

### Experimental

Synthesis experiments were performed using reactive transport employing an ASTeX AX 5310 microwave plasma reactor. Intrinsic (111) oriented silicon substrates were first prepared using standard RCA SC1, HF treatment and SC 2 cleaning procedures to ensure the removal of trace metals, organic compounds and native oxides at the silicon surface. Ga and Sb metals were placed on opposite sides of a graphite susceptor with clean growth substrates placed in between. Iron and quartz were also used as substrates in subsequent experimentation. Hydrogen was bubbled through Di-tert-butyl Phosphine (DTBP) precursor held at 720 torr at a rate of 20 sccm. Hydrogen was used at a flow rate of 200 sccm. The reactor was cycled to a 10^−2^ torr vacuum 3 times then filled with hydrogen stream to an atmosphere of 100 torr. The plasma was then ignited and adjusted to plasma power between 800 and 1000 W. Upon reaching the target plasma power, the DTBP stream was introduced and the plasma reaction was allowed to carry out for 2 hours.

5 individual samples were characterized using X-Ray diffraction (a Bruker Discovery D8 system) utilizing Cu Kα radiation (0.154 nm), transmission electron microscopy (FEI Tecnai F20), and Scanning Electron Microscope (SEM) using an FEI Nova 600 SEM. Diffuse reflectance UV-Vis measurements were performed on each of these samples with a Perkin Elmer Lambda 950 UV-Vis spectrometer with a 60 mm integrating sphere and tauc plot analysis was used to determine band gap. Photoluminescence and Raman measurements were taken from a single sample using a Reneshaw Invia Micro Raman spectrometer with 442 nm laser. For cold PL, a liquid nitrogen cooling stage was used to cool the samples to ~77 K.

The GaP_(1−x)_Sb_x_ samples were cleaned off any unreacted Ga and oxides by dipping in concentrated H_2_SO_4_ (95%–98%) for 120 seconds. Photoelectrochemical testing was performed using a custom three-port cell with a quartz viewing window for illumination connected to a Princeton Applied Research Model 273A Potentiostat/Galvanostat. An electrolyte of 3M H_2_SO_4_ was used as the electrolyte for the three electrode configuration with the GaP_(1−x)_Sb_x_ as working electrode, Platinum mesh as the counter electrode and Accumet Ag/AgCl in 3M NaCl as the reference electrode. IV scans were performed at a scanning rate of 10 mV/s under illumination using a Newport Instruments Oriel 300 W Xe lamp solar simulator with Newport 77330 monochromator that is calibrated using to AM 1.5 using a Thorlabs D10MM power meter with PM100 readout.

## Additional Information

**How to cite this article**: Russell, H. B. *et al.* Direct Band Gap Gallium Antimony Phosphide (GaSb_x_P_1−x_) Alloys. *Sci. Rep.* **6**, 20822; doi: 10.1038/srep20822 (2016).

## Supplementary Material

Supplementary Information

## Figures and Tables

**Figure 1:**
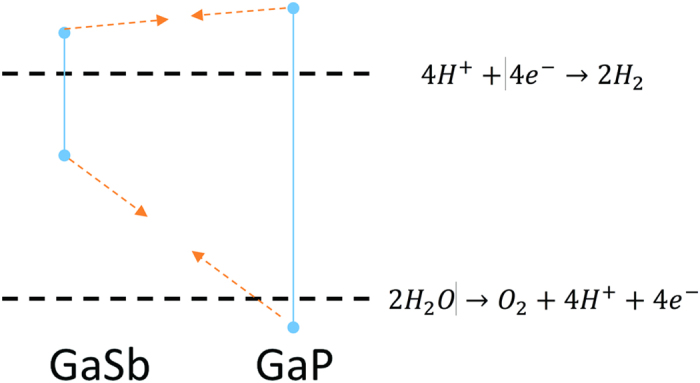
Band positioning of GaSb and GaP and the hydrogen evolution reaction and oxygen evolution reactions at pH = 7.
