# Supplementary material for: Direct Band Gap Gallium Antimony Phosphide (GaSbxP1−x) Alloys
Source: Sci Rep. 2016 Feb 10;6:20822. doi: 10.1038/srep20822 (PMC4748408; doi:10.1038/srep20822)
Supplement: Supplementary Information [file srep20822-s1.pdf]

Supplemental Information

Direct Band Gap Gallium Antimony Phosphide ($GaSb_xP_{1-x}$) Alloys

H.B. Russell,[1] A.N. Andriotis,[3] M. Menon,[2] J. Jasinski,[1] A.M. Garcia[1] and M.K. Sunkara[1,*]

[1]Department of Chemical Engineering and Conn Center for Renewable Energy Research University of Louisville, Louisville, KY, USA
[2]Center for Computational Sciences, University of Kentucky, Lexington, KY, USA

[3]Department of Physics and Astronomy, University of Kentucky, Lexington, KY, USA

[5]Institute of Electronic Structure and Laser (IESL), Foundation of Research and Technology-Hellas (FORTH), Heraklion, Crete, Greece

*mahendra@louisville.edu

Presented in this supplemental information is the energy dispersion x-ray spectroscopy analysis (EDS) confirmation of GaSb at the substrate growth interface, TEM EDS line scan analysis of individual GaSbP NW's and fundamental photoelectrochemical (PEC) characterization of the GaSbP alloy including 3 electrode chopped linear scan voltammetry (IV), open circuit potential (OCP) under chopped illumination and unbiased 2 electrode chronoamperometry.

EDS of GaSbP cross section

A GaSbP sample grown on Si was cleaved and the cross section of the sample was inspected via EDS. As can be seen in supplemental figure 1, in the top section of the sample, Gallium,

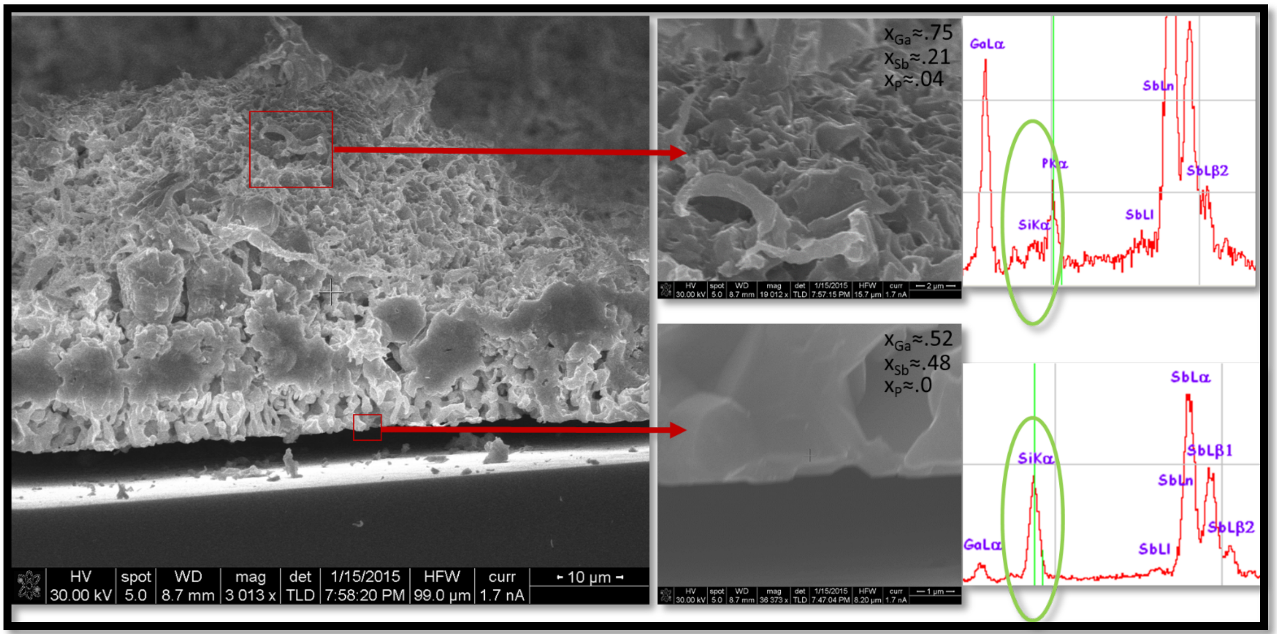

Supplemental Figure 1 – Energy Dispersion X-Ray Spectroscopy of GaSbP cross-section. In the top section showing the surface of the sample there is the presence of phosphorus while in the bottom section there is no phosphorus.

Antimony and Phosphorus are present while at the interface between the silicon substrate and only Ga and Sb are present. This occurs because the phosphorous gas precursor was not introduced into the reactor until the hydrogen plasma had been stabilized at the power chosen for deposition (800-1000W). Thus, initial plasma transport occurred with only the gallium and antimony present in the reaction area, forming GaSb at the interface of our samples as evidenced by the monoatomic ratios of Ga and Sb present at the substrate/growth interface.

TEM EDS Analysis

Line scan and point source EDAX measurements were taken from many GaSbP nanowires in a transmission electron microscope. Compositions determined by EDS show ternary alloys with doping levels up to 19% incorporation of antimony into GaSbP. The similar line scan profiles of Antimony in comparison to Gallium and Phosphorus show uniform incorporation of Antimony and further confirms a ternary alloy composition and not mixed phase GaSb and GaP.

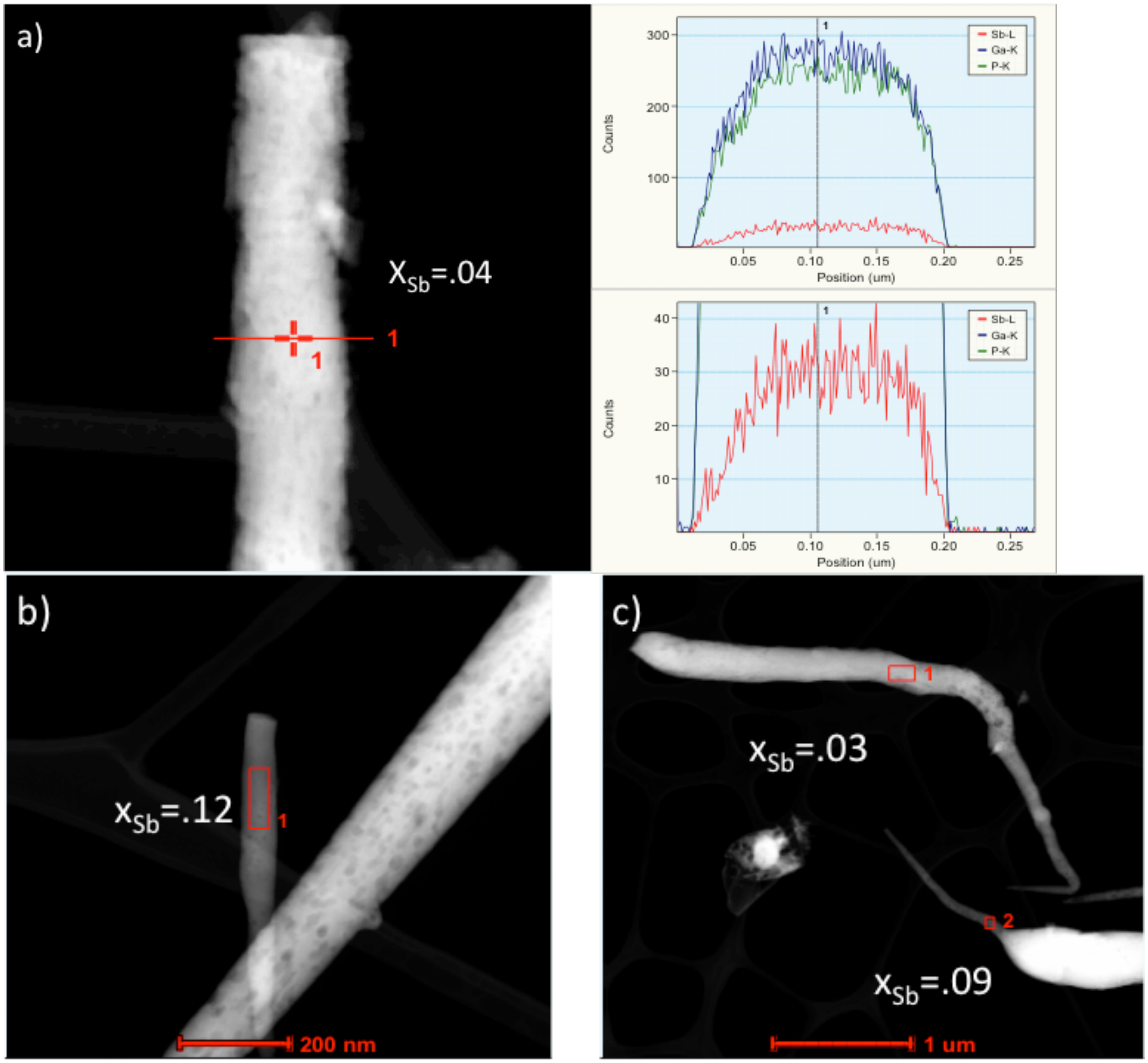

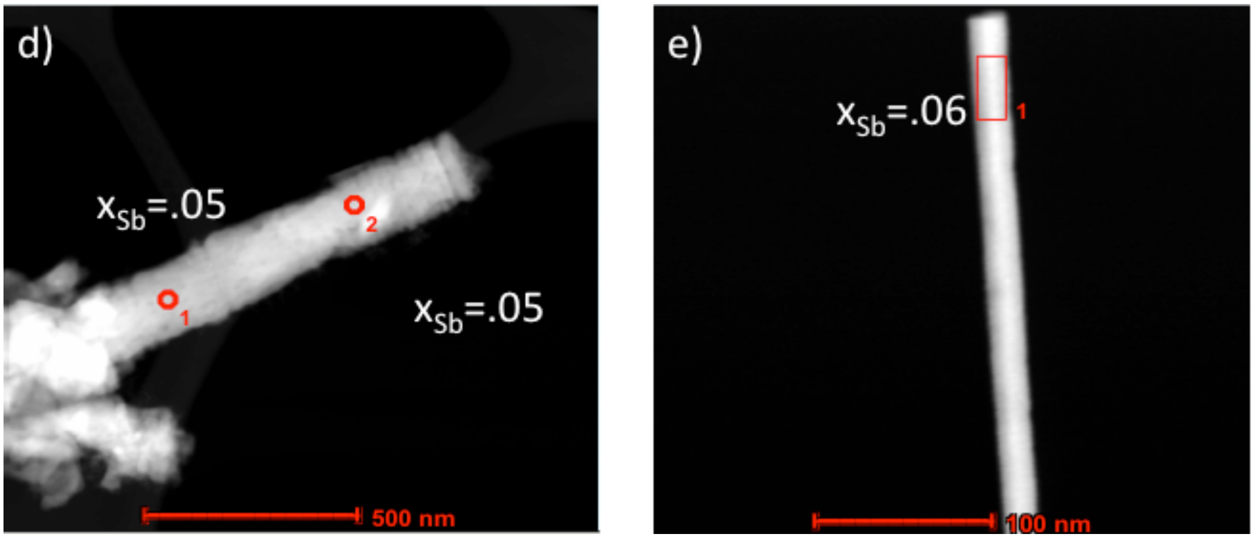

Supplemental Figure 2 – Transmission Electron Microscopy of GaSbP utilizing EDS under both (a) line scan and (b-i) point source conditions.

PHOTOELECTROCHEMISTRY

Preliminary photoelectochemistry for the GaSbP nanowires was performed including 3 electrode linear sweep voltammetry, open circuit potential under chopped illumination and unbiased 2 electrode chronoamperometry. Open circuit potential shows a positive voltage shift under illumination, thus it can be confirmed that the semiconductor exhibits p-type conductivity. 3 Electrode IV measurements show a photoactivity of approximately 40 $\mu A/cm^2$ at -.65 eV vs RHE. Unbiased 2 electrode measurements utilizing a Pt counter electrode were done on our 1.6 eV band gap sample on quartz substrate. A photoactivity of approximately 20 $\mu A/cm^2$ was observed. This is indication that the observed photoactivity is true water splitting by our GaSbP semiconductor.

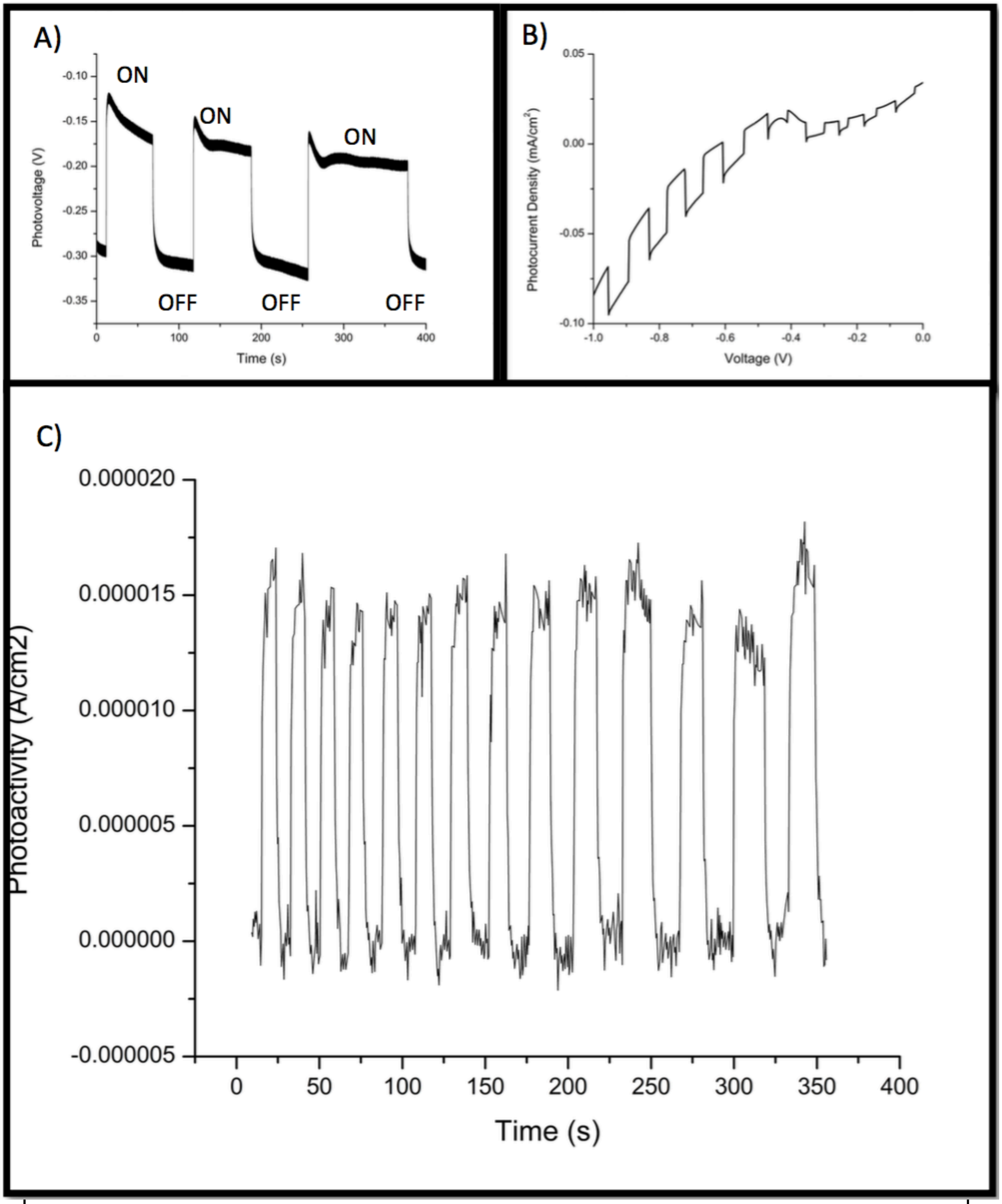

Supplemental Figure 3 – Fundamental photoelectrochemical characterization including linear sweep voltammetry showing an approximate photocurrent density of 40 μA/cm^2 at -.65 eV (a), open circuit potential under chopped illumination indicating p-type conductivity (b) and unbiased 2 electrode chronoamperometry under chopped illumination showing approximately 15 μA/cm^2 photoactivity and confirming true water splitting.
